# Integrating AI Literacy with the TPB-TAM Framework to Explore Chinese University Students’ Adoption of Generative AI

**DOI:** 10.3390/bs15101398

**Published:** 2025-10-15

**Authors:** Xiaoxuan Zhang, Xiaoling Hu, Yinguang Sun, Lu Li, Shiyi Deng, Xiaowen Chen

**Affiliations:** Institute of Moral Education, Central China Normal University, Wuhan 430079, China; zhangxiaoxuan@mails.ccnu.edu.cn (X.Z.); ygsun@mail.ccnu.edu.cn (Y.S.); ulillilu@mails.ccnu.edu.cn (L.L.); dengshiyi@mails.ccnu.edu.cn (S.D.); dawn023@mails.ccnu.edu.cn (X.C.)

**Keywords:** generative artificial intelligence, AI literacy, technology acceptance, Theory of Planned Behavior, Technology Acceptance Model, privacy risk

## Abstract

This study examines Chinese university students’ adoption of generative artificial intelligence (GenAI) tools by integrating the Theory of Planned Behavior (TPB), the Technology Acceptance Model (TAM), and AI literacy dimensions into a hybrid framework. Survey data from 1006 students across various majors and regions are analyzed using partial least squares structural equation modeling. Notably, AI literacy (i.e., students’ AI ethics, evaluation, and awareness) positively affect their attitudes, subjective norms, and perceived behavioral control, although the influence patterns vary according to the literacy dimension. Perceived privacy risks reduce AI trust, which mediates adoption behavior. Overall, core TPB pathways are validated, with behavioral intentions significantly predicting students’ actual use. Gender and regional differences moderate the key relationships. The results of this study suggest that enhancing students’ ethical and evaluative competencies, building user trust, and addressing privacy concerns could promote generative AI integration in education.

## 1. Introduction

The global release of ChatGPT, a generative artificial intelligence (GenAI) tool, in November 2022 transformed the field of education. GenAI tools are reshaping traditional education by enabling personalized learning, enhancing student engagement, and promoting better academic outcomes (W. Dong et al., 2024; Lo et al., 2024; Singh et al., 2025; Yasmin Khairani Zakaria et al., 2025). In China, this shift gained momentum with the launch of the DeepSeek-R1 model in early 2025, which is characterized by high performance, low cost, and its open-source nature (Wei et al., 2025). In particular, education is recognized as a key area where GenAI offers “unique advantages” (Y. Dong et al., 2020), e.g., supporting both student learning and teacher development (Van Den Berg & Du Plessis, 2023). The recent release of China’s Smart Education White Paper underscores the national emphasis on leveraging GenAI in education (MOE, 2025).

Systematic efforts by the Chinese government have prompted universities and private enterprises to integrate GenAI into education, thereby developing application scenarios, such as language learning and intelligent assessments. Thus, China has gradually gained advantages in terms of the practical application of GenAI in education and related research. As a result, the global leadership in GenAI research is shifting from the United States to China, making it particularly meaningful to develop adoption models based on Chinese university students (Knox, 2020; Ma et al., 2024). As GenAI becomes more deeply embedded in academic environments, it is becoming increasingly crucial to understand the factors that influence user acceptance and behaviors. Despite their significant potential in various fields, the rapid proliferation of GenAI tools has raised multiple concerns related to data privacy and ethical risks (Chen & Esmaeilzadeh, 2024), academic integrity (Lo et al., 2024), and user trust (Choudhury & Shamszare, 2023). These concerns highlight the complexity of GenAI adoption, which involves technical feasibility as well as users’ cognitive, emotional, and normative evaluations. To our knowledge, these complexities have not yet been thoroughly explored, especially in the context of Chinese higher education.

Previous studies investigating GenAI adoption in education have typically employed classic frameworks, such as the Technology Acceptance Model (TAM) and the Unified Theory of Acceptance and Use of Technology (UTAUT) (Davis, 1989; Venkatesh et al., 2003). However, few studies have integrated GenAI-specific factors (e.g., ethics, trust, and privacy concerns) into a single composite model for the higher education context. Most either apply classic acceptance frameworks without these variables (Ivanov et al., 2024; Sergeeva et al., 2025) or examine only one factor, such as trust (Nazaretsky et al., 2025). To address these knowledge gaps, the present study investigates the key psychological and behavioral drivers behind GenAI adoption among university students. The Theory of Planned Behavior (TPB) and TAM serve as the core frameworks, which are extended with AI-specific factors. TPB explains behavior according to three cognitive constructs, i.e., Attitudes (ATT), Subjective Norms (SN), and perceived behavioral control, and has been widely adopted in technology acceptance research because of its strong explanatory power (Ajzen, 1991). However, TPB alone does not account for aspects such as AI awareness, trust, ethics, and privacy risks, which are expected to be crucial in GenAI contexts. Therefore, the present study focuses on the following research questions (RQ):

RQ1: What are the key factors that influence Chinese university students’ adoption of GenAI tools?

RQ2: What is the role of AI literacy dimensions in Chinese students’ adoption of GenAI tools?

RQ3: To what extent can the integrated TAM-TPB model explain Chinese students’ use of GenAI tools?

## 2. Literature Review

### 2.1. Theoretical Model Evolution

Understanding individuals’ behavioral responses to emerging technologies is a core pillar of technology adoption research. Traditional models, such as the TAM (Davis, 1989), TPB (Ajzen, 1991), and UTAUT (Venkatesh et al., 2003), provide a theoretical basis for explaining users’ technology adoption intentions and behaviors. However, the rapid development and wide application of AI technologies have brought new challenges related to ethical, psychological, and situational aspects. In general, traditional models have difficulty encompassing these complexities.

The TAM emphasizes perceived usefulness and perceived ease of use, and this approach is widely used in various technological environments (Davis, 1989). However, AI systems differ significantly from traditional information systems. For example, they are autonomous, rely on probabilistic decision-making, and often exhibit “black box” characteristics (Ribeiro et al., 2016). As a result, factors related to trust (Glikson & Woolley, 2020), ethical issues, privacy risks (Dwivedi et al., 2021), and algorithmic transparency (Shin, 2021) have emerged as key variables affecting user adoption of GenAI. The TPB introduces subjective norms and perceived behavioral control, while emphasizing the influence of social expectations and self-efficacy beliefs on behavioral intentions. In the context of GenAI adoption, this theory was extended to incorporate more cognitive and affective dimension variables, such as perceived algorithmic fairness (Shin, 2020), affective trust (Glikson & Woolley, 2020), and AI attributes (Al-Emran et al., 2024). The UTAUT model builds on these foundational models by incorporating additional variables, such as support conditions and social influence (Venkatesh et al., 2012; Vorm & Combs, 2022). Nevertheless, all of these models require adaptation when applied to GenAI contexts. Recent research has indicated that when developing intentions to use GenAI, users focus on performance expectations and effort expectations but also consider the autonomy of AI, ethical consistency, and possible negative consequences (Shin & Park, 2019).

To accommodate the unique attributes of AI, researchers have constructed hybrid models that incorporate traditional adoption variables as well as AI-specific variables. Common AI-specific variables include Awareness of AI (AWA), Ethics of AI (ETH), Evaluation of AI (EVA), AI Trust (AIT), and Perceived Privacy Risk (PPR). These are not ancillary supplements to the core constructs, but rather, they represent key factors for understanding how users accept smart technology. Meanwhile, Behavioral Intentions (BI) and Use Behavior (UB) remain the primary outcome variables, inheriting the predictive logic of traditional models and mediating the effects of other variables. Recent theoretical developments have revealed a trend toward context-sensitive and trust-oriented models. For example, scholars advocate the introduction of new dimensions, such as perceived algorithmic justice and AI literacy (Floridi et al., 2018; Mittelstadt et al., 2016), to reflect the public’s attitudes toward AI services in high-risk scenarios, such as healthcare, finance, and education.

In summary, traditional models emphasize technology usefulness, ease of use, and social influence, but in the context of GenAI adoption, additional factors such as trust and ethics must also be considered. Therefore, the conceptual model proposed herein integrates classical predictors (e.g., ATT, SN, BI, UB) with GenAI-specific variables (e.g., AWA, EVA, ETH, AIT, PPR) to offer a comprehensive framework for explaining user adoption behaviors in the era of smart technologies.

### 2.2. Model Development

The conceptual model developed in this study integrates traditional constructs from established adoption theories with GenAI-specific variables. The resulting framework acknowledges that user acceptance of GenAI is shaped by classical cognitive and social determinants, as well as the ethics, trust, and privacy concerns inherent to AI technologies. Specifically, the comprehensive model is grounded in foundational models (e.g., TAM, TPB, UTAUT) and informed by recent advances in GenAI adoption research, which have guided hypothesis development.

#### 2.2.1. AI Literacy

In classic models, such as TAM and TPB, users’ ATT, BI, and UB are limited by traditional variables (Ajzen, 1991; Davis, 1989). However, with highly complex and dynamically evolving technologies, such as AI, users must have basic cognition and judgment before they can adopt these tools (Moravec et al., 2024). Therefore, AWA, ETH, and EVA are considered key cognitive dimensions of AI literacy (Ng et al., 2021; B. Wang et al., 2023), which have been embedded in the original model framework. Although previous frameworks (B. Wang et al., 2023) commonly include “usage” as a dimension of AI literacy, it was not incorporated in the present study because it overlaps conceptually with the outcome variable of UB. Including “usage” as both a predictor and an outcome would create conceptual redundancy and statistical endogeneity, which could compromise the interpretability of the structural model and results (Antonakis et al., 2010). Therefore, only the cognitive dimensions of AWA, ETH, and EVA were retained to avoid violating model specification principles.

AWA refers to the user’s ability to recognize and understand the application of AI in various areas of daily life. Specifically, it is reflected in an individual’s ability to correctly identify which tools, services, or devices use AI technology (Moravec et al., 2024). Higher AWA enhances the user’s sense of control over the technology, while helping reduce psychological resistance to its use (U.S. Department of Education, 2023). Individuals with higher AWA are more likely to develop positive attitudes toward the use of such technology because they can proactively identify the necessary resources to support them and respond sensitively to mainstream adoption trends (Chan & Hu, 2023).

ETH refers to an individual’s understanding of the ethical principles, social impacts, and moral responsibilities of AI, which guide AI development, application, and governance (Floridi et al., 2018). Therefore, if users perceive AI systems as ethically sound, their attitudes toward using such technology will improve. They will also tend to place greater trust in external support resources and respond more positively to the subjective norms of GenAI use. 

EVA refers to an individual’s ability to analyze, select, and critically evaluate AI-generated data and information, including the capacity to judge the functions and limitations of generative AI systems and to make informed decisions about their appropriate use in specific contexts (C. Wang et al., 2025). The ethical scenario analysis involved in the assessment strengthens students’ sense of social responsibility and motivates them to consider potential ethical risks when using AI. A positive evaluation of GenAI directly reinforces the user’s attitudes toward GenAI use and boosts their confidence in the platform’s support. It can also promote the user’s acceptance of others using GenAI, thereby enhancing subjective norms (Ng et al., 2021). Although these dimensions are related, they serve different cognitive roles in students’ engagement with AI; AWA involves understanding what AI is, EVA focuses on judging how reliable and useful it is, and ETH addresses whether it is appropriate and responsible to use it.

Based on the theoretical logic described above, the following hypotheses are proposed:

**H1.**  *Awareness of AI positively influences users’ Attitudes Toward using Technology (AWA → ATT).*

**H2.**  *Awareness of AI positively influences users’ Perceived Behavioral Control (AWA → PBC).*

**H3.**  *Awareness of AI positively influences users’ perceived Subjective Norms (AWA → SN).*

**H4.**  *The perceived Ethics of AI positively influence user Attitudes Toward using Technology (ETH → ATT).*

**H5.**  *The perceived Ethics of AI positively influence user Perceived Behavioral Control (ETH → PBC).*

**H6.**  *The perceived Ethics of AI positively influence user perceived Subjective Norms (ETH → SN).*

**H7.**  *The Evaluation of AI positively influences user Attitudes Toward using Technology (EVA → ATT).*

**H8.**  *The Evaluation of AI positively influences user Perceived Behavioral Control (EVA → PBC).*

**H9.**  *The Evaluation of AI positively influences user perceived Subjective Norms (EVA → SN).*

#### 2.2.2. AI Trust and Perceived Privacy Risk 

Considering the highly complex and unpredictable nature of AI, it is critical for users to have mechanisms for trusting AI systems. AIT refers to an individual’s willingness to endure vulnerability when using an AI system based on the expectation that the AI will autonomously perform a specific important action, without their monitoring or control (Glikson & Woolley, 2020). AIT makes users more willing to accept and tolerate aspects of the GenAI tool that are not transparent. In contrast, a lack of trust heightens users’ sensitivity to issues, which can trigger negative evaluations or even rejection of GenAI. Providing transparency can help promote user trust and reinforce positive attitudes, whereas a lack of transparency may erode trust and worsen attitudes (Mittelstadt et al., 2016). When users trust GenAI to be professionally competent, predictable, and free of malicious intent, their attitudes toward GenAI use will be more positive; typically, they are also more willing to accept technical services provided by the platform, which can enhance their perception of the resources (Venkatesh et al., 2003). In addition, AIT shapes user attitudes by influencing risk perception and emotions. Specifically, high trust reduces sensitivity to risks, making users more willing to accept and rely on AI despite ethical flaws. In contrast, low trust increases user alertness to bias and opacity, leading to resistance, abandonment, or negative feedback. At the group level, high trust fosters a shared belief that AI is safe to use, whereas low trust generates collective skepticism, potentially slowing ethical regulation and technology adoption (Colquitt et al., 2007; Mittelstadt et al., 2016).

PPR refers to users’ perceived risks related to the potential that AI systems could lead to privacy breaches, misuse of personal information, or loss of control during data collection, usage, and storage (Y. Zhang et al., 2021). In sensitive domains, such as healthcare and finance, even a small possibility of personal data leakage dramatically weakens users’ trust in AI systems, thereby reducing their acceptance of AI-driven services (Shin, 2020; Y. Zhang et al., 2021). Data privacy and protection constitute fundamental conditions for sustaining user trust and enabling technology adoption. The United States population has expressed mixed attitudes toward AI; although many see the need for careful oversight, their top concerns include data privacy breaches, AI-assisted surveillance, cyber-attacks, and misinformation (B. Zhang & Dafoe, 2019). Such concerns, particularly those related to privacy, are linked to individuals’ trust in AI systems. Therefore, high PPR is expected to undermine AI trust. However, rather than directly discouraging adoption, privacy concerns typically erode users’ trust in AI systems, which in turn lowers their willingness to adopt such tools (Beldad et al., 2010; Pavlou, 2003). Therefore, PPR is considered an antecedent of AIT, rather than a direct predictor of BI in this model.

Based on the above discussion, the following hypotheses are proposed:

**H10.**  *Perceived Privacy Risk negatively affects AI Trust (PPR → AIT).*

**H11.**  *AI Trust positively affects users’ Attitudes Toward using Technology (AIT → ATT).*

**H12.**  *AI Trust positively affects users’ Perceived Behavioral Control (AIT → PBC).*

**H13.**  *AI Trust positively affects users’ perceived Subjective Norms (AIT → SN).*

#### 2.2.3. Behavioral Motivation-Attitude, Support, and Subjective Norms

ATT, PBC, and SN constitute the internal and external drivers of GenAI use intentions. These variables serve as core mediators or antecedent variables in the TAM, TPB, and UTAUT models and dictate behavioral intentions at the cognitive, emotional, and social levels (Ajzen, 1991; Davis, 1989; Venkatesh et al., 2003).

ATT reflects an individual’s overall evaluation and tendency toward a GenAI system (Ajzen, 1991; Davis, 1989). These are mainly based on its perceived usefulness and perceived ease of use, which manifest as emotional and behavioral tendencies to accept or resist the use of the system (Davis, 1989). Users’ attitudes toward GenAI are the psychological outcome of their combined cognition, trust, and performance expectations. Cognition influences performance perceptions through trust, which ultimately drives behaviors (e.g., continued use) through emotion and satisfaction, with the attitude variable being the psychological cornerstone (Shin, 2020). 

PBC refers users’ perceptions regarding whether the AI system has the necessary resources, services, and environmental safeguards (Venkatesh et al., 2003). A well-developed support system can help remove technical barriers and increase self-confidence, especially for users with non-technical backgrounds (Venkatesh et al., 2012). 

SN reflects whether users perceive expectations from significant others or organizations. This factor represents an important manifestation of social pressures at the behavioral level (Ajzen, 1991). Because GenAI technology has not yet become ubiquitous, GenAI adoption is particularly affected by social trends and group behaviors. For example, in healthcare and education, the adoption of AI systems by mainstream organizations can be expected to significantly increase individuals’ willingness to use AI tools (Shin & Park, 2019).

Thus, the following hypotheses are proposed:

**H14.**  *Attitudes Toward using Technology positively influence Behavioral Intentions (ATT → BI).*

**H15.**  *Perceived Behavioral Control positively influences Behavioral Intentions (PBC → BI).*

**H16.**  *Subjective Norms positively influence Behavioral Intentions (SN → BI).*

#### 2.2.4. Behavioral Intentions and Use Behavior

Although users may form strong technology adoption intentions on a psychological level, multiple internal and external factors dictate whether these intentions can be transformed into actual usage behaviors. BI is a psychological representation of a user’s subjective willingness to use a GenAI tool, whereas UB is the concrete action that translates these intentions into reality (Venkatesh et al., 2003). Studies have shown that BI is the most reliable predictor of UB (Oliveira et al., 2016). In a context where GenAI has not yet been fully institutionalized and deployed, users’ inclinations to translate “willingness to use” into “actual use” is a key measure of adoption success.

Therefore, the following hypothesis is proposed:

**H17.**  *Behavioral Intentions positively influence actual Use Behavior (BI → UB).*

**Figure 1:**
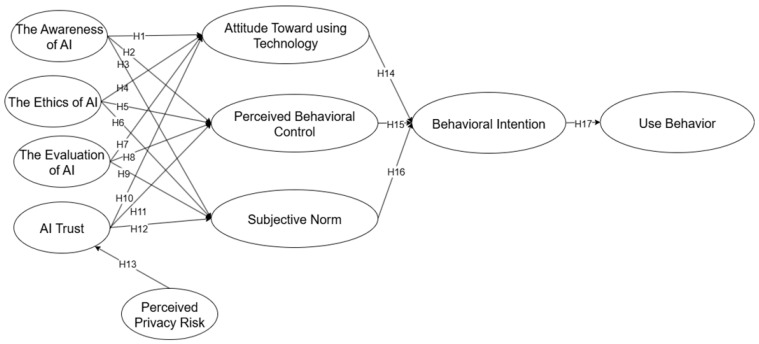
Research model.

The proposed research model, which summarizes all the hypotheses (H1–H17), is shown in Figure 1.

## 3. Methodology

### 3.1. Survey Design 

During questionnaire development, multiple validated scales from the literature were considered. UB was measured as an observed variable based on participants’ self-reported actual frequency of GenAI use, and other constructs were treated as latent variables.

Because attitudes toward technology can change quickly, this study used an online questionnaire to rapidly collect relevant data from a large sample, to minimize transcription errors, and to increase data collection efficiency (Regmi et al., 2016). However, online questionnaires face notable challenges. For example, it is difficult to ensure data validity, and financial incentives may reduce response credibility or increase duplicate responses (Wright, 2005). To motivate participation, monetary incentives were provided in this study during the data collection stage. Additionally, multiple data quality control measures were implemented to ensure the validity and reliability of the data. Specifically, the questionnaire could be completed only one time on a single device to avoid repeated responses, and polygraph questions were included to identify and exclude invalid or randomly answered questionnaires. Ultimately, 1006 valid questionnaire responses were obtained.

The questionnaire had two parts. The first part collected demographic data (e.g., gender, age, major) to understand participants’ backgrounds. The second part covered GenAI usage, technology acceptance variables, and related factors (Table 1). Before completing the survey, participants were given a concise definition of GenAI, referencing local Chinese tools like DeepSeek (https://chat.deepseek.com/) and Doubao (https://www.doubao.com/chat/), to ensure consistent baseline understanding across the sample population. For abstract variables, such as AWA, BI, and PPR, context-specific questions were used to improve measurement accuracy. The SN construct measured social pressure from parents, teachers, and classmates, which are all key influencers in Chinese society. The final survey questionnaire comprised 28 items measuring 9 latent variables each on a 5-point Likert scale (1 = Strongly Disagree, 5 = Strongly Agree).

### 3.2. Sampling and Data Collection Procedures

In April 2025, the survey was provided to university students nationwide using the online data collection platform Wenjuan Xing (https://www.wjx.cn/). At this time, students had resumed campus life, which helped ensure the authenticity of the data. The questionnaire link was distributed to students across multiple universities, inviting voluntary participation with monetary rewards as incentives; the sample may exhibit some self-selection bias due to this recruitment approach. A total of 1701 college students completed the questionnaire. After excluding 693 responses that were flagged for invalidity (e.g., containing inconsistent answers to lie detection items), 1006 valid responses remained for analysis and model construction.

In terms of gender distribution, females comprised the majority 764 participants (75.94%), indicating a notable gender imbalance. Additionally, the sample included mostly individuals who were 18 to 23 years old, accounting for 90.36% (909 participants), consistent with the typical demographic of college students. Participants aged 24 to 29 years constituted 8.55% (86 participants), and participants aged 30 and above corresponded to only 1.10% (11 participants). In terms of academic majors, liberal arts students were the largest group (671 participants; 66.70%), followed by engineering students (159 participants; 15.81%), science students (106 participants; 10.54%), and other majors (70 participants; 6.96%). Geographically, the study covered the Eastern, Southern, Western, Northern, and Central regions of China. However, there was an uneven distribution, with Central China representing 68.19%, while Southern and Northern China accounted for only 1.69% and 2.09%, respectively (Table 2).

Overall, the dataset underrepresented male students, students majoring in science and engineering, and students from Northern and Southern China. Follow-up studies in our laboratory will aim to obtain a more balanced distribution of participants across gender, academic majors, and regions to more accurately reflect the relevant population. These demographic imbalances (e.g., 75.94% female, 68.19% from Central China) may have introduced bias in group difference analyses because the overrepresentation of certain groups might have amplified or weakened some pathway relationships. Therefore, multi-group analyses by gender and region were conducted (refer to Section 4.2 and Section 5.4) to statistically assess and partially address this potential bias.

### 3.3. Data Analysis

This study aimed to construct a structural model with ATT, SN, and PBC as mediating variables, BI and UB as key dependent variables, and AI literacy dimensions (AWA, EVA, ETH, AIT, and PPR) as exogenous variables. Given the model’s complexity and the study’s emphasis on prediction-oriented exploration rather than strict theory confirmation, Partial Least Squares Structural Equation Modeling (PLS-SEM) was adopted as the analytical approach. Hair et al. (2019) demonstrated that PLS-SEM is particularly appropriate when the research focuses on theory development or predicting key target constructs, rather than simply confirming established models.

Furthermore, PLS-SEM is robust in handling models with numerous indicators and latent variables, especially under non-normal data distributions, formative-reflective construct combinations, and moderate sample sizes (Fong & Law, 2013; Sarstedt et al., 2014). This method has undergone continuous development and can manage many data variables, nonlinear relationships, and irrelevant laws, making it a flexible and generalizable tool in contemporary behavioral and information system research (Wold et al., 2001).

The SmartPLS 4 program was employed to conduct the PLS-SEM analysis. All measurement and structural model assessments indicated that the data met reliability and validity standards, and the model fit indices fell within acceptable thresholds, suggesting that the proposed structural model has good explanatory power and matches the empirical data well.

## 4. Results

### 4.1. Validity and Reliability Tests

To examine the measurement quality of the scales, the PLS-SEM model was used for data analysis. In the reflective measurement module, the confirmatory composite analysis (CCA) assessments included the outer loading and significance, indicator reliability, combined reliability, average variance extracted (AVE), discriminant validity (heterotrait–monotrait; HTMT), nomological validity, and predictive validity, with an emphasis on reliability and validity assessments based on shared variance (Hair et al., 2020). The outer loading results ranged from 0.752 to 1, thus satisfying the threshold requirement of ≥0.708 (Table 3) (Hair et al., 2011). Cronbach’s alpha values ranged from 0.739 to 0.941, all exceeding the required threshold of ≥0.7 (Hair & Sarstedt, 2019). Rho-A was between 0.771 and 0.945, all greater than the necessary 0.7 (Hair et al., 2019). Composite reliability was between 0.863 and 0.973, and AVE results were between 0.678 and 0.923, thus all meeting the basic requirements of the data reliability (Table 4) (Hair et al., 2020). These results confirmed that all measurement instruments were statistically reliable and valid, providing a sound basis for the structural model analysis.

The HTMT is useful for revealing sensitivity and specificity (Henseler et al., 2015), and all HTMT ratios were less than 0.9, indicating no issues regarding discriminant validity (Table 5) (Gold et al., 2001; T. S. H. Teo et al., 2008). Together, these findings indicate that the measurement scales are consistent, valid, and distinct from one another, providing a sound foundation for structural model testing.

### 4.2. Structural Model Evaluation

The key components of the structural model evaluation included the tests of covariance (VIF), explanatory power (R^2^), and effect size (f^2^). The VIF should be ≤3; f^2^ values of 0.02, 0.15, and 0.35 represent small, medium, and large effect sizes, respectively; and R^2^ reflects the extent to which the model explains the variance of the endogenous construct, with 0.75, 0.50, and 0.25 corresponding to significant, moderate, and weak explanatory power, respectively (Hair et al., 2011, 2019).

The VIF results for the model ranged from 1.000 to 1.838, all of which were less than the threshold of 3, indicating no covariance issues (Table 6). Most hypothesized pathways had f^2^ effect sizes greater than 0.25 (many even exceeding 0.75), indicating medium-to-large effects. Only two pathways, i.e., AWA → SN and EVA → ATT, had f^2^ values less than 0.25. Therefore, consistent with the non-significant bootstrapping results, these weak-effect pathways (AWA → SN and EVA → ATT) were excluded from the final model. To clarify this decision, their exclusion was justified theoretically. First, SN reflects social pressures from significant others (e.g., parents, teachers, peers), which are externally shaped and not directly influenced by individual cognitive abilities, such as AWA. According to the reasoned action approach, SN are formed primarily by normative beliefs about important others’ expectations, whereas awareness or knowledge serves as a background factor that indirectly affects beliefs (Fishbein & Ajzen, 2011). Therefore, awareness alone is unlikely to directly alter established social norms. Second, university students—especially digital natives—tend to form stable attitudes toward GenAI based on emotional predispositions, peer culture, and media narratives, rather than rational evaluations (Brewer et al., 2022; Katsantonis & Katsantonis, 2024). Thus, EVA is more likely to affect PBC and SN than ATT. These null effects likely reflect underlying cognitive and socio-cultural mechanisms and not methodological flaws.

**Figure 2:**
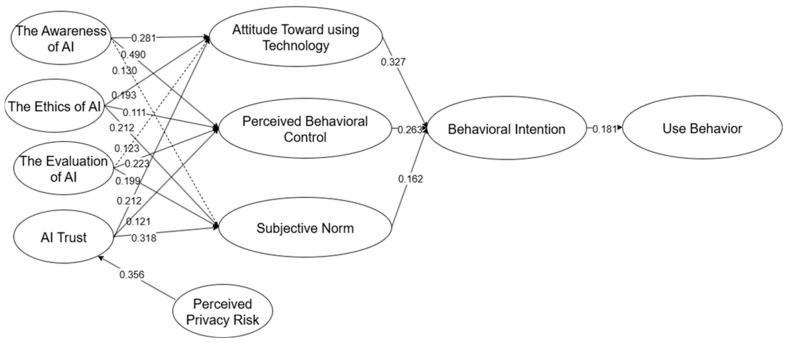
Final model with path coefficients. **Note:** Solid lines mean significant, and dotted lines mean not significant.

Overall, these results indicate that the structural model explains a substantial proportion of variance in the key constructs, supporting most hypothesized paths and thus validating the proposed model. The final validated model with path coefficients is presented in Figure 2.

The bootstrapping method in PLS-SEM was used to analyze the relationships between the variables involved in the model. Bootstrapping is a nonparametric resampling method that does not require strict distributional assumptions, is widely applicable, and is easily understood (Streukens & Leroi-Werelds, 2016). The results revealed the following: AIT was significant for ATT (β = 0.212, *p* < 0.001), PBC (β = 0.121, *p* < 0.001), SN (β = 0.318, *p* < 0.001), and BI (β = 0.327, *p* < 0.001); AWA influenced both ATT (β = 0.281, *p* < 0.001) and PBC (β = 0.490, *p* < 0.001); ETH had an effect on ATT (β = 0.193, *p* < 0.001), PBC (β = 0.111, *p* < 0.001), and SN (β = 0.212, *p* < 0.001); EVA did not affect ATT but did significantly impact PBC (β = 0.223, *p* < 0.001) and SN (β = 0.199, *p* < 0.001). Furthermore, the pathways BI → UB (β = 0.181, *p* < 0.001), PBC → BI (β = 0.263, *p* < 0.001), and SN → BI (β = 0.162, *p* < 0.001) were all consistent with the TPB model, and PPR → AIT (β = 0.356, *p* < 0.001) was consistent with the TAM (Table 7).

To further explore potential group differences in the model, this study included group analysis by gender to compare the performance of males and females in each pathway relationship. Although most pathways reached statistical significance in both gender groups (*p* < 0.05), several pathways differed appreciably in terms of significance and strength. Specifically, the pathways AIT → PBC (β = 0.134, *p* < 0.001), ETH → SN (β = 0.219, *p* < 0.001), EVA → PBC (β = 0.209, *p* = 0.003), and EVA → SN (β = 0.212, *p* < 0.001) were significant in the female sample population but did not exhibit the same level of statistical significance among the male participants. These results suggest that females are more likely than males to develop a sense of behavioral control and identify with subjective norms when influenced by attitudes, moral perceptions, and environmental values. In addition, the pathways ATT → BI, PBC → BI, PPR → AIT, and SN → BI showed significant and positive effects in both genders, suggesting that the mechanisms for forming behavioral intentions have consistent structures. Overall, the findings indicate that gender moderates certain relationships in the proposed model. Future research could further examine these differences by considering gender-related psychological mechanisms and socio-cultural factors.

Multi-group analyses were also conducted by region, revealing notable differences in the direction and magnitude of certain pathway coefficients across different regions of China. In the Southern region, significance test results could not be obtained due to the small sample size; nevertheless, the degree of association between variables may be relatively strong in terms of the path coefficients. For example, the path coefficients of AIT → SN (β = 0.713), AWA → PBC (β = 0.897), and PBC → BI (β = 0.521) are all higher in the South, suggesting that in this region, the public may be more prone to the effects of perceived trustworthiness and convenience, which may result in stronger behavioral intentions. The Northern region also lacked the support of significance indicators, but some of the path directions were informative. For example, AIT → PBC (β = −0.188) and ETH → ATT (β = −0.120) were in a negative direction, indicating that there may be different cognitive modes or attitude tendencies affecting the mechanisms of influence between variables in this region relative to other regions. Meanwhile, some of the pathway coefficients were higher in the Northern region, e.g., EVA → ATT (β = 0.569) and PBC → BI (β = 0.738), reflecting more pronounced public attitudes and behavioral responses under the influence of these variables. In contrast, the Eastern region had complete significance test results, with multiple pathways, including AIT → PBC (*p* = 0.338), AWA → ATT (*p* = 0.054), and ETH → ATT (*p* = 0.168) failing the significance test, suggesting relatively weak influential relationships between these variables. The central region exhibited more stable and generalized pathway relationships, with AIT → ATT, AWA → PBC, and PBC → BI having moderate coefficients and high significance, reflecting balanced inter-variable interactions. The Western region is characterized by “strong local correlation and diversified overall structure”; for example, AIT → ATT (*p* = 0.304) and AWA → PBC (*p* = 0.423) are strong paths, whereas PPR → AIT (*p* = 0.289) and BI → UB (*p* = 0.208) have relatively weak effects. These results highlight meaningful gender and regional differences, suggesting that social and cultural factors shape how students respond to AI-related variables.

Overall, the data were in strict accordance with the needs of the study, as well as the corresponding standards and requirements.

## 5. Discussion 

This study empirically examined the factors influencing Chinese college students’ adoption of GenAI by integrating the TPB and TAM frameworks with AI literacy dimensions. The findings confirm the applicability of the TPB core pathways, while revealing the nuanced roles of AI literacy dimensions (AWA, EVA, ETH) and perceived privacy risk in shaping trust and behavioral intentions. Group difference analyses further demonstrated significant variations across gender and regions. 

### 5.1. TPB 

TPB was proposed by Ajzen in 1985 as an extension of the Theory of Reasoned Action (TRA). This model considers behavioral beliefs, normative beliefs, and control beliefs as the bases for the formation of attitudes, subjective norms, and perceived behavioral control, respectively, which collectively predict behavioral intentions (Ajzen, 1985, 1991). In the model developed in the present study, four variables were used: ATT, SN, PBC, and BI. The results were generally consistent with those obtained from the TPB model.

Based on the results, it is reasonable to conclude that attitudes toward GenAI (β = 0.327) affect Chinese college students’ behavioral intentions, which in turn affect their behaviors. In other words, if college students have positive attitudes toward GenAI, they will be more inclined to use GenAI-powered tools. Similarly, perceived social pressure (β = 0.162) affects college students’ use of technology, and PBC has the greatest effect on students’ behavioral intentions and actual behaviors (β = 0.263). These results are also consistent with previous research. For example, Cheon et al. studied m-learning among American students and found that ATT, SN, and PBC all positively influenced m-learning adoption intentions; moreover, they observed that perceived behavioral control had the greatest impact, followed by attitudes and subjective norms (Cheon et al., 2012). According to Ajzen (2002), PBC comprises self-efficacy and controllability. Its strong predictive effect on behavioral intentions stems largely from the self-efficacy aspect, which reflects an individual’s confidence in performing the behavior. Because PBC integrates internal and external control factors related to the behavior’s feasibility, it has a more direct influence on intentions (i.e., motivations) than attitudes or subjective norms (Ajzen, 2002).

### 5.2. The Influence of AI Literacy on GenAI Adoption

AI literacy was first validated using structural equation modeling by Chai et al. who demonstrated the influence of AI literacy on middle school students’ attitudes toward learning AI. The authors proposed that AI literacy comprises three dimensions: understanding, application, and evaluation (Chai et al., 2020). Later, B. Wang et al. (2023) developed an operational measurement tool including an AI literacy scale based on four dimensions: awareness, usage, evaluation, and ethics (B. Wang et al., 2023). The present study adopted the latter report’s dimensional framework. Because usage was already considered as a dependent variable, only the dimensions of awareness, evaluation, and ethics were included as AI literacy dimensions. Among these AI literacy variables, ETH significantly influenced attitudes, SN, and PBC. Meanwhile, AWA had no effect on SN, likely because SN reflects social pressures, which depend on external environments, whereas AWA is an individual capability and cannot directly alter established social consensus. Thus, the influence of SN stems from “social environments” rather than individual cognitive levels (Achmadi et al., 2025). EVA had no impact on ATT—this finding contradicts prior studies, which reported that EVA significantly influenced ATT, and the design of evaluative dimensions affected the reliability of attitude measurements (Grassini, 2023; Stein et al., 2024). A plausible explanation is that, as digital natives, students are frequently exposed to AI in daily life, leading to stable attitudes shaped more by peer culture, media narratives, and emotional predispositions than by rational evaluations. In addition, the evaluative dimensions assessed in this study may not have fully captured the issues most salient to students, thereby weakening the connection with attitudes. A ceiling effect may also exist because students generally hold optimistic views of AI, which reduces the incremental impact of further evaluations. Finally, disciplinary differences and sample composition could have obscured weak effects. Together, these discrepancies may be explained by the impacts of participant characteristics, evaluative design, and attitude formation mechanisms, indicating that for university students, EVA shapes PBC and SN, rather than directly influencing ATT.

Previous research that integrated TPB with AI literacy showed that among the AI literacy dimensions, ethics scored the highest, and awareness scored the lowest (C. Wang et al., 2025) This is consistent with the results of the present study, which showed that ETH scored the highest (M = 4.05), indicating that students were concerned about the ethical risks of GenAI. Meanwhile, EVA (M = 3.68) and AWA (M = 3.52) scored lower. This was likely because AI technologies have already been deeply integrated into daily life and learning scenarios; however, students tend to “use it daily without knowing it” and do not actively pay attention to or explore the principles behind these AI technologies (C. Wang et al., 2025). This type of “unconscious use” keeps students’ overall knowledge of AI technology at a surface functional level. However, they tend to lack an in-depth understanding of its underlying operating mechanism, technical characteristics, and other core knowledge, leading to relatively weak AI awareness literacy.

### 5.3. TAM 

In China’s digital economy, massive user behavior data and highly concentrated platform ecosystems contain behavioral traces that are weakly identifiable yet strongly correlated, and thus, fall outside the current identifiability-based personal information protection framework. This gap leads to heightened risks of privacy leaks, algorithmic manipulation, and data monopolies (Shang, 2025). Therefore, the present study adopted the TAM to probe Chinese users’ perceived privacy risks regarding AI platforms’ processing of their digital behavioral traces. The primary focus was the relationship between perceived privacy risks and AI trust.

The empirical analysis indicated that these perceived privacy risks had a significant negative impact on participants’ AI trust. This is consistent with previous research that has identified perceived privacy risks as an important negative predictor of AI trust. Some studies have even highlighted its prominence in the education sector (Gumusel, 2025; C. Zhang et al., 2025). Although in this study PPR was modeled only as an antecedent of AI trust, future research could also examine PPR as a direct predictor of behavioral intention, considering the increasing global concerns about data privacy.

### 5.4. Group Difference Analysis

These gender differences may be related to socially constructed gender role expectations, differences in risk perception, and variations in academic background distribution. Women are often socialized to place greater emphasis on adhering to norms and ethical responsibilities and tend to be more sensitive to the potential risks of new technologies. Therefore, when forming subjective norms and perceived behavioral control, they are more likely to be influenced by ethical concerns and evaluative judgments. In contrast, men may be more inclined toward instrumental and outcome-oriented motivations for technology use. These underlying mechanisms may help explain why women showed stronger significance in the AIT → PBC, ETH → SN, EVA → PBC, and EVA → SN pathways.

Various factors could also account for the observed regional differences. The South and North represent more economically or politically dynamic regions, which might exhibit faster or more extreme responses to new information. The Central region’s relatively balanced development corresponds to more stable user behavior patterns. In contrast, the West may be constrained by a digital divide, while the East could be experiencing information overload. Each of these considerations could limit or distort the influence of certain factors. These regional differences emphasize the importance of considering regional characteristics when formulating strategies to improve the precision of policy and behavioral interventions. The reasons for the observed geographical differences may include regional development levels, digital infrastructure development, population education levels, policy guidance strength, and socio-cultural differences. Additionally, the Southern and Northern regions were not supported by statistical significance in this study due to the limited subsample sizes. Thus, the relevant conclusions are mainly based on the numerical trends of the path coefficients and should be interpreted with caution. Future studies should further verify the characteristics of the regional differences and explore them in depth by analyzing larger samples and optimizing the data collection strategy to enhance robustness and generalizability.

## 6. Theoretical Contributions and Practical Implications

### 6.1. Theoretical Contributions

This study makes important theoretical contributions by enriching TPB-based research on AI literacy (C. Wang et al., 2025) through a new perspective of AI literacy. Specifically, AI literacy is reconceptualized as comprising three prerequisite cognitive dimensions (i.e., AWA, ETH, and EVA), while treating “usage” as an outcome but not a component. This shifts from viewing AI literacy as skills developed through usage to seeing it as cognitive readiness that precedes behavioral decision-making. Furthermore, unlike traditional models emphasizing rational evaluations of usefulness and ease of use (Davis, 1989; Venkatesh et al., 2003), this study highlights the foundational role of cognitive readiness in shaping university students’ behavioral responses to AI.

Building on this theoretical foundation, the present study makes two key theoretical contributions. First, an integrated model was constructed by combining TPB and TAM, and incorporating AWA, EVA, ETH, AIT, and PPR. By embedding these emerging AI literacy constructs into the classical TPB-TAM framework, the developed model exhibits enhanced explanatory power in the context of GenAI adoption. This comprehensive structure addresses limitations of traditional models by accounting for users’ cognitive, ethical, and trust-related concerns related to AI usage.

Second, two distinct psychological pathways through which AI literacy influences BI are revealed, i.e., a cognitive–social pressure pathway (ETH → SN → BI) and a cognitive–self-efficacy pathway (AWA → PBC → BI). These pathways clarify how AI literacy dimensions contribute to users’ perceived social expectations and behavioral confidence, thereby enriching the TPB’s behavioral prediction mechanism in AI contexts.

### 6.2. Practical Implications

From a practical standpoint, this study demonstrates that PPR has a significant negative impact on AIT, which reduces BI. This mediating effect of AIT suggests that improving data protection mechanisms and enhancing transparency in AI systems can foster trust and promote adoption. To achieve this, platform developers could introduce clear consent prompts, privacy dashboards, and accessible data-use policies, and universities could establish data governance guidelines to enhance institutional accountability. Meanwhile, policymakers should strengthen legal frameworks to ensure platform compliance with privacy protection standards. The results presented herein suggest that Chinese university students exhibit strong ETH but relatively low AWA. This imbalance indicates the need for tiered AI literacy education, such as embedding introductory AI concepts and ethical principles earlier and offering advanced modules about critical evaluation and risk assessment in later academic years. This approach would ensure coverage across different majors and academic levels. For example, universities could embed AI literacy into existing curricula by incorporating ethical debates in humanities courses, case-based evaluation exercises in computer science classes, or interdisciplinary seminars that allow students to critically assess AI tools in practice.”

Additionally, group analysis shows gender- and region-based differences. For example, females demonstrated stronger pathway relationships for AIT → PBC, ETH → SN, EVA → PBC, and EVA → SN, indicating a greater sensitivity to ethical and evaluative factors in shaping SN and PBC. However, this heightened sensitivity may also make them more cautious and risk-averse when adopting new AI tools. Therefore, mentorship programs and tailored learning resources can provide trusted guidance and reduce perceived risks, helping students translate ethical concerns into positive adoption behaviors. Regional analysis revealed heterogeneous pathway strengths and directions across Eastern, Central, Western, Northern, and Southern China, reflecting possible socio-cultural and infrastructural disparities. These differences highlight the need for regionally adaptive policies and targeted support measures (e.g., mentorship programs or specialized learning resources) for female students or students from less digitally developed regions.

In conclusion, this study advances theoretical development by enriching TPB and TAM with cognitive, ethical, and trust/privacy dimensions. In doing so, it provides actionable insights for promoting GenAI adoption through policy, education, and platform design tailored to diverse user groups.

## 7. Limitations and Future Prospects

This study expands the understanding of GenAI attitudes and behavioral intentions by integrating the TPB-TAM framework with AI literacy dimensions; however, some limitations remain. First, sample representativeness is constrained. Despite nationwide survey distribution, 68.06% of respondents were from Central China, 75% were female, and 57.9% were humanities and social sciences majors, which limited the generalizability of the findings across regions, genders, and disciplines. As shown in the multi-group analyses, gender and regional differences moderated certain pathways. Therefore, the demographic imbalance (e.g., more females and more respondents from Central China) may have systematically influenced the strength of some relationships, which should be considered when interpreting the findings. Future studies should employ stratified analysis or quota sampling strategies to obtain more balanced samples and enhance the external validity of the results. Second, the cross-sectional design captures attitudes and intentions at a single point in time, but this approach makes it difficult to track how attitudes, trust, and behaviors evolve as GenAI continues its rapid development. Future research should adopt longitudinal tracking to observe changes over time and strive for more balanced samples across regions, genders, and disciplines to increase the generalizability of the results and conclusions.

## 8. Conclusions

This study developed an integrated model of factors influencing college students’ GenAI usage by building upon TPB and TAM through the incorporation of three AI literacy dimensions. Results from a nationwide survey showed that ATT, SN, and PBC all positively affected behavioral intentions in terms of AI usage. Among AI literacy factors, EVA had the widest influence, significantly affecting ATT, SN, and PBC. AWA influenced ATT and PBC, but not SN, suggesting that awareness alone does not shift subjective norms. ETH had no significant effect on ATT, possibly due to differences in academic backgrounds and application contexts. The model also incorporated PPR as an extension of the TAM to reflect China’s digital platform environment. PPR negatively affected AIT, which mediated its impact on behavioral intentions and actual use, thus highlighting the importance of privacy protections and trust-building.

Recommendations based on the results reported herein include the following. First, students’ AI evaluation skills should be enhanced so they can better understand tools and their limitations. Second, contextualized AI ethics education tailored to specific disciplines should be provided. Third, platform privacy protections and transparency should be improved. Finally, legal safeguards for digital behavior data should be strengthened, as well as requirements for interpretability and user control. 

Overall, this study advances the theoretical and empirical understanding of GenAI acceptance, thereby offering guidance for technology promotion and policy in higher education. Future research efforts should adopt longitudinal designs and evaluate cross-platform, cross-cultural samples to increase the generalizability and applicability of the findings.

## Figures and Tables

**Table 1:** Measurement scale.

Construct	Code	Question	Source
AIT	AIT1	The functionality of GenAI is reliable.	(Al-Emran et al., 2022)
	AIT2	GenAI can be trusted.	
	AIT3	I believe it is feasible to use GenAI in learning activities.	
ATT	ATT1	GenAI makes learning and working more interesting.	(T. Teo, 2016; Venkatesh et al., 2003)
	ATT2	I can accept the idea of using GenAI.	
	ATT3	Using GenAI is a good idea.	
AWA	AWA1	I can distinguish between GenAI systems and non-GenAI systems.	(Calvani et al., 2009; C. Wang et al., 2025)
	AWA2	I know how GenAI systems can help me.	
	AWA3	I can identify the GenAI technologies adopted in the applications and products I use.	
BI	BI1	In the future, I plan to continue using GenAI.	(Taylor & Todd, 1995; Venkatesh et al., 2003)
	BI2	I will keep trying to use GenAI in my daily life.	
	BI3	I plan to continue using GenAI frequently.	
ETH	ETH1	I always follow ethical principles when using GenAI.	(Calvani et al., 2009; C. Wang et al., 2025)
	ETH2	I am very concerned about privacy and information security issues when using GenAI.	
	ETH3	I try not to abuse GenAI.	
EVA	EVA1	I can evaluate the functions and limitations of GenAI systems after using them for a period of time.	
	EVA2	I can choose the appropriate solutions from the various solutions provided by GenAI systems.	
	EVA3	I can select the most suitable GenAI system for a specific task from various GenAI systems.	
PBC	PBC1	I have control over using GenAI.	(Morris & Venkatesh, 2000)
	PBC2	I have the resources necessary to use GenAI.	
	PBC3	I have the knowledge necessary to use GenAI.	
PPR	PPR1	During my personal learning process and in future teaching, I am worried that GenAI will collect too much of my personal information.	(C. Zhang et al., 2025)
	PPR2	During my personal learning process and in future teaching, GenAI will use my personal information for other purposes without my authorization.	
	PPR3	During my personal learning process and in future teaching, GenAI will share my personal information without my authorization.	
SN	SN1	My parents support me in learning how to use GenAI.	(Venkatesh et al., 2012; C. Wang et al., 2025)
	SN2	My teacher believes it is necessary to learn how to use GenAI.	
	SN3	My classmates think it is necessary to learn how to use GenAI.	
	SN4	Most of the people I know think I should know how to use GenAI.	

AI = Artificial Intelligence; AIT = AI Trust; ATT = Attitude Toward using Technology; AWA = Awareness of AI; BI = Behavioral Intention; ETH = Ethics of AI; EVA = Evaluation of AI; PBC = Perceived Behavioral Control; PPR = Perceived Privacy Risk; SN = Subjective Norms; UB = Use Behavior.

**Table 2:** Demographic characteristics of respondents (N = 1006).

Attributes	Attributes	Frequency	Percentage (%)
Gender	Male	242	24.06
	Female	764	75.94
Age	18–23 years old	909	90.36
	24–29 years old	86	8.55
	30–35 years old	6	0.60
	≥36 years old	5	0.50
Major	Liberal Arts	671	66.70
	Science	106	10.54
	Engineering	159	15.81
	Others	70	6.96
Education	Associate’s degree	217	21.57
	Bachelor’s degree	673	66.90
	Master’s degree	96	9.54
	Doctoral degree	23	2.29
Region	West China	114	11.33
	East China	168	16.70
	Central China	686	68.19
	South China	17	1.69
	North China	21	2.09

**Table 3:** Results of cross-loading.

	AIT	ATT	AWA	BI	ETH	EVA	PBC	PPR	SN
AIT1	**0.858**	0.375	0.429	0.364	0.346	0.412	0.431	0.301	0.438
AIT2	**0.822**	0.293	0.357	0.269	0.259	0.374	0.346	0.269	0.394
AIT3	**0.832**	0.527	0.373	0.448	0.481	0.512	0.471	0.318	0.584
ATT1	0.452	**0.922**	0.507	0.512	0.376	0.456	0.520	0.209	0.439
ATT2	0.456	**0.916**	0.437	0.519	0.427	0.436	0.520	0.211	0.515
ATT3	0.494	**1.000**	0.515	0.561	0.436	0.485	0.566	0.228	0.518
AWA1	0.299	0.283	**0.745**	0.220	0.161	0.343	0.451	0.181	0.293
AWA2	0.457	0.551	**0.849**	0.476	0.385	0.557	0.654	0.215	0.453
AWA3	0.330	0.354	**0.820**	0.321	0.201	0.455	0.538	0.234	0.339
BI1	0.433	0.562	0.425	**0.939**	0.415	0.474	0.510	0.231	0.477
BI2	0.403	0.494	0.408	**0.942**	0.349	0.441	0.490	0.216	0.421
BI3	0.443	0.558	0.442	**1.000**	0.403	0.484	0.530	0.237	0.474
ETH1	0.429	0.439	0.311	0.419	**0.906**	0.413	0.395	0.228	0.463
ETH2	0.373	0.362	0.305	0.341	**0.882**	0.405	0.355	0.223	0.411
ETH3	0.372	0.335	0.243	0.293	**0.829**	0.381	0.351	0.279	0.401
EVA1	0.458	0.391	0.492	0.389	0.344	**0.839**	0.489	0.313	0.422
EVA2	0.458	0.454	0.474	0.445	0.402	**0.869**	0.521	0.287	0.471
EVA3	0.429	0.389	0.504	0.401	0.417	**0.838**	0.546	0.277	0.477
PBC1	0.387	0.495	0.570	0.454	0.398	0.529	**0.810**	0.215	0.441
PBC2	0.471	0.487	0.587	0.460	0.321	0.509	**0.869**	0.243	0.470
PBC3	0.406	0.432	0.579	0.414	0.335	0.492	**0.822**	0.256	0.437
PPR1	0.330	0.223	0.183	0.232	0.368	0.321	0.265	**0.803**	0.345
PPR2	0.288	0.172	0.243	0.174	0.151	0.264	0.218	**0.861**	0.261
PPR3	0.251	0.161	0.225	0.173	0.132	0.254	0.212	**0.804**	0.292
SN1	0.488	0.419	0.385	0.343	0.363	0.392	0.427	0.329	**0.752**
SN2	0.466	0.391	0.352	0.363	0.398	0.436	0.430	0.297	**0.848**
SN3	0.489	0.472	0.378	0.430	0.464	0.473	0.473	0.304	**0.886**
SN4	0.498	0.451	0.429	0.451	0.408	0.497	0.473	0.303	**0.859**

**Note:** The bold values indicate the highest factor loadings of each indicator on its corresponding construct. AI = Artificial Intelligence; AIT = AI Trust; ATT = Attitude Toward using Technology; AWA = Awareness of AI; BI = Behavioral Intention; ETH = Ethics of AI; EVA = Evaluation of AI; PBC = Perceived Behavioral Control; PPR = Perceived Privacy Risk; SN = Subjective Norms; UB = Use Behavior.

**Table 4:** Measurement model.

Construct	Item	Outer Loading	Cronbach’s Alpha (α > 0.7)	Rho-A (>0.7)	Composite Reliability (>0.7)	AVE (>0.5)
AIT	AIT1	0.858	0.792	0.811	0.876	0.701
	AIT2	0.822				
	AIT3	0.832				
ATT	ATT1	0.922	0.941	0.945	0.963	0.896
	ATT2	0.916				
	ATT3	1.000				
AWA	AWA1	0.745	0.736	0.772	0.847	0.649
	AWA2	0.849				
	AWA3	0.820				
BI	BI1	0.939	0.958	0.960	0.973	0.923
	BI2	0.942				
	BI3	1.000				
ETH	ETH1	0.906	0.844	0.853	0.906	0.762
	ETH2	0.882				
	ETH3	0.829				
EVA	EVA1	0.839	0.806	0.808	0.886	0.721
	EVA2	0.869				
	EVA3	0.838				
PBC	PBC1	0.810	0.781	0.781	0.873	0.696
	PBC2	0.869				
	PBC3	0.822				
PPR	PPR1	0.803	0.764	0.771	0.863	0.678
	PPR2	0.861				
	PPR3	0.804				
SN	SN1	0.752	0.857	0.863	0.904	0.702
	SN2	0.848				
	SN3	0.886				
	SN4	0.859				

AI = Artificial Intelligence; AIT = AI Trust; ATT = Attitude Toward using Technology; AWA = Awareness of AI; BI = Behavioral Intention; ETH = Ethics of AI; EVA = Evaluation of AI; PBC = Perceived Behavioral Control; PPR = Perceived Privacy Risk; SN = Subjective Norms; UB = Use Behavior.

**Table 5:** Results of heterotrait–monotrait ratio analysis.

	AIT	ATT	AWA	BI	ETH	EVA	PBC	PPR	SN	UB
AIT										
ATT	0.549									
AWA	0.586	0.588								
BI	0.492	0.590	0.499							
ETH	0.526	0.487	0.389	0.447						
EVA	0.645	0.556	0.726	0.551	0.554					
PBC	0.629	0.660	0.893	0.614	0.518	0.771				
PPR	0.447	0.265	0.350	0.273	0.330	0.433	0.364			
SN	0.684	0.577	0.563	0.523	0.572	0.645	0.658	0.450		
UB	0.117	0.118	0.165	0.185	0.028	0.095	0.109	0.042	0.123	

AI = Artificial Intelligence; AIT = AI Trust; ATT = Attitude Toward using Technology; AWA = Awareness of AI; BI = Behavioral Intention; ETH = Ethics of AI; EVA = Evaluation of AI; PBC = Perceived Behavioral Control; PPR = Perceived Privacy Risk; SN = Subjective Norms; UB = Use Behavior.

**Table 6:** Results of variance inflation factor analysis.

	AIT	ATT	AWA	BI	ETH	EVA	PBC	PPR	SN	UB
AIT		1.567					1.567		1.567	
ATT				1.623						
AWA		1.580					1.580		1.580	
BI										1.000
ETH		1.370					1.370		1.370	
EVA		1.838					1.838		1.838	
PBC				1.697						
PPR	1.000									
SN				1.573						
UB										

AI = Artificial Intelligence; AIT = AI Trust; ATT = Attitude Toward using Technology; AWA = Awareness of AI; BI = Behavioral Intention; ETH = Ethics of AI; EVA = Evaluation of AI; PBC = Perceived Behavioral Control; PPR = Perceived Privacy Risk; SN = Subjective Norms; UB = Use Behavior.

**Table 7:** Summary of hypothesis testing.

Hypothesis	Path Coefficient (β) (Bootstrap Mean)	Effect Size (f2)	T Statistics (|O/STDEV|)	*p* Values	Conclusion
AIT → ATT	0.212	0.047	6.535	0.000	Supported
AIT → PBC	0.121	0.023	4.245	0.000	Supported
AIT → SN	0.318	0.118	9.532	0.000	Supported
ATT → BI	0.327	0.110	8.869	0.000	Supported
AWA → ATT	0.281	0.083	7.857	0.000	Supported
AWA → PBC	0.490	0.378	15.734	0.000	supported
AWA → SN	0.130	0.020	3.741	0.000	Unsupported
BI → UB	0.181	0.034	5.306	0.000	Supported
ETH → ATT	0.193	0.045	5.507	0.000	Supported
ETH → PBC	0.111	0.023	3.791	0.000	Supported
ETH → SN	0.212	0.060	7.195	0.000	Supported
EVA → ATT	0.123	0.014	3.245	0.001	Unsupported
EVA → PBC	0.223	0.067	6.508	0.000	Supported
EVA → SN	0.199	0.040	5.468	0.000	Supported
PBC → BI	0.263	0.068	7.188	0.000	Supported
PPR → AIT	0.356	0.146	9.186	0.000	Supported
SN → BI	0.162	0.028	4.653	0.000	Supported

AI = Artificial Intelligence; AIT = AI Trust; ATT = Attitude Toward using Technology; AWA = Awareness of AI; BI = Behavioral Intention; ETH = Ethics of AI; EVA = Evaluation of AI; PBC = Perceived Behavioral Control; PPR = Perceived Privacy Risk; SN = Subjective Norms; UB = Use Behavior.

## Data Availability

The data presented in this study are not publicly available because they will be used for follow-up longitudinal research. To protect participant privacy and to avoid compromising ongoing data collection, the dataset is temporarily restricted. Data may be made available from the corresponding author on reasonable request.

## References

[B1-behavsci-15-01398] Achmadi H., Samuel S., Patria D. (2025). The influence of AI literacy, subjective norm, attitude toward using AI, perceived usefulness of AI and confidence of learning AI toward behavior intention. International Journal of Educational Research and Development.

[B2-behavsci-15-01398] Ajzen I., Kuhl J., Beckmann J. (1985). From intentions to actions: A theory of planned behavior. Action Control.

[B3-behavsci-15-01398] Ajzen I. (1991). The theory of planned behavior. Organizational Behavior and Human Decision Processes.

[B4-behavsci-15-01398] Ajzen I. (2002). Perceived behavioral control, self-efficacy, locus of control, and the theory of planned behavior. Journal of Applied Social Psychology.

[B5-behavsci-15-01398] Al-Emran M., Abu-Hijleh B., Alsewari A. A. (2024). Exploring the effect of Generative AI on social sustainability through integrating AI attributes, TPB, and T-EESST: A deep learning-based hybrid SEM-ANN approach. IEEE Transactions on Engineering Management.

[B6-behavsci-15-01398] Al-Emran M., Al-Maroof R., Al-Sharafi M. A., Arpaci I. (2022). What impacts learning with wearables? An integrated theoretical model. Interactive Learning Environments.

[B7-behavsci-15-01398] Antonakis J., Bendahan S., Jacquart P., Lalive R. (2010). On making causal claims: A review and recommendations. The Leadership Quarterly.

[B8-behavsci-15-01398] Beldad A., De Jong M., Steehouder M. (2010). How shall I trust the faceless and the intangible? A literature review on the antecedents of online trust. Computers in Human Behavior.

[B9-behavsci-15-01398] Brewer P. R., Bingaman J., Paintsil A., Wilson D. C., Dawson W. (2022). Media use, interpersonal communication, and attitudes toward artificial intelligence. Science Communication.

[B10-behavsci-15-01398] Calvani A., Fini A., Ranieri M. (2009). Assessing digital competence in secondary education—Issues, models and instruments.

[B11-behavsci-15-01398] Chai C. S., Wang X., Xu C. (2020). An extended theory of planned behavior for the modelling of Chinese secondary school students’ intention to learn artificial intelligence. Mathematics.

[B12-behavsci-15-01398] Chan C. K. Y., Hu W. (2023). Students’ voices on generative AI: Perceptions, benefits, and challenges in higher education. International Journal of Educational Technology in Higher Education.

[B13-behavsci-15-01398] Chen Y., Esmaeilzadeh P. (2024). Generative AI in medical practice: In-depth exploration of privacy and security challenges. Journal of Medical Internet Research.

[B14-behavsci-15-01398] Cheon J., Lee S., Crooks S. M., Song J. (2012). An investigation of mobile learning readiness in higher education based on the theory of planned behavior. Computers & Education.

[B15-behavsci-15-01398] Choudhury A., Shamszare H. (2023). Investigating the impact of user trust on the adoption and use of ChatGPT: Survey analysis. Journal of Medical Internet Research.

[B16-behavsci-15-01398] Colquitt J. A., Scott B. A., LePine J. A. (2007). Trust, trustworthiness, and trust propensity: A meta-analytic test of their unique relationships with risk taking and job performance. Journal of Applied Psychology.

[B17-behavsci-15-01398] Davis F. D. (1989). Perceived usefulness, perceived ease of use, and user acceptance of information technology. MIS Quarterly.

[B18-behavsci-15-01398] Dong W., Pan D., Kim S. (2024). Exploring the integration of IoT and generative AI in English language education: Smart tools for personalized learning experiences. Journal of Computational Science.

[B19-behavsci-15-01398] Dong Y., Hou J., Zhang N., Zhang M. (2020). Research on how human intelligence, consciousness, and cognitive computing affect the development of artificial intelligence. Complexity.

[B20-behavsci-15-01398] Dwivedi Y. K., Hughes L., Ismagilova E., Aarts G., Coombs C., Crick T., Duan Y., Dwivedi R., Edwards J., Eirug A., Galanos V., Ilavarasan P. V., Janssen M., Jones P., Kar A. K., Kizgin H., Kronemann B., Lal B., Lucini B., Williams M. D. (2021). Artificial Intelligence (AI): Multidisciplinary perspectives on emerging challenges, opportunities, and agenda for research, practice and policy. International Journal of Information Management.

[B21-behavsci-15-01398] Fishbein M., Ajzen I. (2010). Predicting and changing behavior.

[B22-behavsci-15-01398] Floridi L., Cowls J., Beltrametti M., Chatila R., Chazerand P., Dignum V., Luetge C., Madelin R., Pagallo U., Rossi F., Schafer B., Valcke P., Vayena E. (2018). AI4People—An ethical framework for a good AI society: Opportunities, risks, principles, and recommendations. Minds and Machines.

[B23-behavsci-15-01398] Fong L., Law R. (2013). Hair, J. F. Jr., Hult, G. T. M., Ringle, C. M., Sarstedt, M. (2014). A primer on partial least squares structural equation modeling (PLS-SEM). Sage Publications. ISBN: 978-1-4522-1744-4. 307 pp. European Journal of Tourism Research.

[B24-behavsci-15-01398] Glikson E., Woolley A. W. (2020). Human trust in artificial intelligence: Review of empirical research. Academy of Management Annals.

[B25-behavsci-15-01398] Gold A. H., Malhotra A., Segars A. H. (2001). Knowledge management: An organizational capabilities perspective. Journal of Management Information Systems.

[B26-behavsci-15-01398] Grassini S. (2023). Development and validation of the AI attitude scale (AIAS-4): A brief measure of general attitude toward artificial intelligence. Frontiers in Psychology.

[B27-behavsci-15-01398] Gumusel E. (2025). A literature review of user privacy concerns in conversational chatbots: A social informatics approach: An annual review of information science and technology (ARIST) paper. Journal of the Association for Information Science and Technology.

[B29-behavsci-15-01398] Hair J. F., Howard M. C., Nitzl C. (2020). Assessing measurement model quality in PLS-SEM using confirmatory composite analysis. Journal of Business Research.

[B30-behavsci-15-01398] Hair J. F., Ringle C. M., Sarstedt M. (2011). PLS-SEM: Indeed a silver bullet. Journal of Marketing Theory and Practice.

[B31-behavsci-15-01398] Hair J. F., Risher J. J., Sarstedt M., Ringle C. M. (2019). When to use and how to report the results of PLS-SEM. European Business Review.

[B28-behavsci-15-01398] Hair J. F., Sarstedt M. (2019). Factors versus composites: Guidelines for choosing the right structural equation modeling method. Project Management Journal.

[B32-behavsci-15-01398] Henseler J., Ringle C. M., Sarstedt M. (2015). A new criterion for assessing discriminant validity in variance-based structural equation modeling. Journal of the Academy of Marketing Science.

[B33-behavsci-15-01398] Ivanov S., Soliman M., Tuomi A., Alkathiri N. A., Al-Alawi A. N. (2024). Drivers of generative AI adoption in higher education through the lens of the Theory of Planned Behaviour. Technology in Society.

[B34-behavsci-15-01398] Katsantonis A., Katsantonis I. G. (2024). University students’ attitudes toward artificial intelligence: An exploratory study of the cognitive, emotional, and behavioural dimensions of AI attitudes. Education Sciences.

[B35-behavsci-15-01398] Knox J. (2020). Artificial intelligence and education in China. Learning, Media and Technology.

[B36-behavsci-15-01398] Lo C. K., Hew K. F., Jong M. S. (2024). The influence of ChatGPT on student engagement: A systematic review and future research agenda. Computers & Education.

[B37-behavsci-15-01398] Ma D., Akram H., Chen H. (2024). Artificial intelligence in higher education: A cross-cultural examination of students’ behavioral intentions and attitudes. The International Review of Research in Open and Distributed Learning.

[B38-behavsci-15-01398] Ministry of Education of the People’s Republic of China (2025). China’s smart education white paper.

[B39-behavsci-15-01398] Mittelstadt B. D., Allo P., Taddeo M., Wachter S., Floridi L. (2016). The ethics of algorithms: Mapping the debate. Big Data & Society.

[B40-behavsci-15-01398] Moravec V., Hynek N., Gavurova B., Kubak M. (2024). Everyday artificial intelligence unveiled: Societal awareness of technological transformation. Oeconomia Copernicana.

[B41-behavsci-15-01398] Morris M. G., Venkatesh V. (2000). Age differences in technology adoption decisions: Implications for a changing work force. Personnel Psychology.

[B42-behavsci-15-01398] Nazaretsky T., Mejia-Domenzain P., Swamy V., Frej J., Käser T. (2025). The critical role of trust in adopting AI-powered educational technology for learning: An instrument for measuring student perceptions. Computers and Education: Artificial Intelligence.

[B43-behavsci-15-01398] Ng D. T. K., Leung J. K. L., Chu K. W. S., Qiao M. S. (2021). AI literacy: Definition, teaching, evaluation and ethical issues. Proceedings of the Association for Information Science and Technology.

[B44-behavsci-15-01398] Oliveira T., Thomas M., Baptista G., Campos F. (2016). Mobile payment: Understanding the determinants of customer adoption and intention to recommend the technology. Computers in Human Behavior.

[B45-behavsci-15-01398] Pavlou (2003). Consumer acceptance of electronic commerce: Integrating trust and risk with the technology acceptance model. International Journal of Electronic Commerce.

[B46-behavsci-15-01398] Regmi P. R., Waithaka E., Paudyal A., Simkhada P., van Teijlingen E. (2016). Guide to the design and application of online questionnaire surveys. Nepal Journal of Epidemiology.

[B47-behavsci-15-01398] Ribeiro M. T., Singh S., Guestrin C. (2016). “Why should I trust you?”: Explaining the predictions of any classifier. The 22nd ACM SIGKDD International Conference on Knowledge Discovery and Data Mining.

[B48-behavsci-15-01398] Sarstedt M., Ringle C. M., Smith D., Reams R., Hair J. F. (2014). Partial least squares structural equation modeling (PLS-SEM): A useful tool for family business researchers. Journal of Family Business Strategy.

[B49-behavsci-15-01398] Sergeeva O. V., Zheltukhina M. R., Shoustikova T., Tukhvatullina L. R., Dobrokhotov D. A., Kondrashev S. V. (2025). Understanding higher education students’ adoption of generative AI technologies: An empirical investigation using UTAUT2. Contemporary Educational Technology.

[B50-behavsci-15-01398] Shang X. (2025). Legal orientation of digital behavioral traces and the protection of rights and interests: Addressing the personal information protection challenges for AI platforms such as DeepSeek. Jinan Journal (Philosophy & Social Sciences).

[B51-behavsci-15-01398] Shin D. (2020). User perceptions of algorithmic decisions in the personalized AI system: Perceptual evaluation of fairness, accountability, transparency, and explainability. Journal of Broadcasting & Electronic Media.

[B52-behavsci-15-01398] Shin D. (2021). The effects of explainability and causability on perception, trust, and acceptance: Implications for explainable AI. International Journal of Human-Computer Studies.

[B53-behavsci-15-01398] Shin D., Park Y. J. (2019). Role of fairness, accountability, and transparency in algorithmic affordance. Computers in Human Behavior.

[B54-behavsci-15-01398] Singh A. K., Kiriti M. K., Singh H., Shrivastava A. (2025). Education AI: Exploring the impact of artificial intelligence on education in the digital age. International Journal of System Assurance Engineering and Management.

[B55-behavsci-15-01398] Stein J.-P., Messingschlager T., Gnambs T., Hutmacher F., Appel M. (2024). Attitudes towards AI: Measurement and associations with personality. Scientific Reports.

[B56-behavsci-15-01398] Streukens S., Leroi-Werelds S. (2016). Bootstrapping and PLS-SEM: A step-by-step guide to get more out of your bootstrap results. European Management Journal.

[B57-behavsci-15-01398] Taylor S., Todd P. A. (1995). Understanding information technology usage: A test of competing models. Information Systems Research.

[B58-behavsci-15-01398] Teo T. (2016). Modelling Facebook usage among university students in Thailand: The role of emotional attachment in an extended technology acceptance model. Interactive Learning Environments.

[B59-behavsci-15-01398] Teo T. S. H., Srivastava S. C., Jiang L. (2008). Trust and electronic government success: An empirical study. Journal of Management Information Systems.

[B60-behavsci-15-01398] U.S. Department of Education, Office of Educational Technology (2023). Artificial intelligence and the future of teaching and learning: Insights and recommendations.

[B61-behavsci-15-01398] Van Den Berg G., Du Plessis E. (2023). ChatGPT and Generative AI: Possibilities for its contribution to lesson planning, critical thinking and openness in teacher education. Education Sciences.

[B62-behavsci-15-01398] Venkatesh V., Morris M. G., Davis G. B., Davis F. D. (2003). User acceptance of information technology: Toward a unified view. MIS Quarterly.

[B63-behavsci-15-01398] Venkatesh V., Thong J. Y. L., Xu X. (2012). Consumer acceptance and use of information technology: Extending the unified theory of acceptance and use of technology. MIS Quarterly.

[B64-behavsci-15-01398] Vorm E. S., Combs D. J. Y. (2022). Integrating transparency, trust, and acceptance: The intelligent systems technology acceptance model (ISTAM). International Journal of Human–Computer Interaction.

[B65-behavsci-15-01398] Wang B., Rau P.-L. P., Yuan T. (2023). Measuring user competence in using artificial intelligence: Validity and reliability of artificial intelligence literacy scale. Behaviour & Information Technology.

[B66-behavsci-15-01398] Wang C., Wang H., Li Y., Dai J., Gu X., Yu T. (2025). Factors influencing university students’ behavioral intention to use generative artificial intelligence: Integrating the theory of planned behavior and AI literacy. International Journal of Human–Computer Interaction.

[B67-behavsci-15-01398] Wei J., Jia K., Zeng R., He Z., Qiu L., Yu W., Tang M., Huang H., Zeng X., Zhang H., Zheng L., Zhang H., Zhang X., Zhao J., Fu H., Jiang Y. (2025). Artificial intelligence innovation development and governance transformation under the DeepSeek breakthrough effect. E-Government.

[B68-behavsci-15-01398] Wold S., Trygg J., Berglund A., Antti H. (2001). Some recent developments in PLS modeling. Chemometrics and Intelligent Laboratory Systems.

[B69-behavsci-15-01398] Wright K. B. (2005). Researching internet-based populations: Advantages and disadvantages of online survey research, online questionnaire authoring software packages, and web survey services. Journal of Computer-Mediated Communication.

[B70-behavsci-15-01398] Yasmin Khairani Zakaria N., Hashim H., Azhar Jamaludin K. (2025). Exploring the impact of AI on critical thinking development in ESL: A systematic literature review. Arab World English Journal.

[B71-behavsci-15-01398] Zhang B., Dafoe A. (2019). Artificial intelligence: American attitudes and trends. SSRN Electronic Journal.

[B72-behavsci-15-01398] Zhang C., Hu M., Wu W., Kamran F., Wang X. (2025). Unpacking perceived risks and AI trust influences pre-service teachers’ AI acceptance: A structural equation modeling-based multi-group analysis. Education and Information Technologies.

[B73-behavsci-15-01398] Zhang Y., Wu M., Tian G. Y., Zhang G., Lu J. (2021). Ethics and privacy of artificial intelligence: Understandings from bibliometrics. Knowledge-Based Systems.

